# Playful pigs: Evidence of consistency and change in play depending on litter and developmental stage

**DOI:** 10.1016/j.applanim.2017.09.018

**Published:** 2018-01

**Authors:** S.M. Brown, R. Peters, I.M. Nevison, A.B. Lawrence

**Affiliations:** a University of Edinburgh, Roslin Institute, Penicuik, EH25 9RG, United Kingdom; b SRUC, West Mains Road, Edinburgh, EH9 3JG, United Kingdom; c Biomathematics and Statistics Scotland, James Clerk Maxwell Building, Peter Guthrie Tait Road, Edinburgh EH9 3FD, United Kingdom

**Keywords:** Play, Piglet, Litter differences, Weaning, Growth

## Abstract

Play behaviour in pre-weaned piglets has previously been shown to vary consistently between litters. This study aimed to determine if these pre-weaning litter differences in play behaviour were also consistent in the post-weaning period. Seven litters of commercially bred piglets were raised in a free farrowing system (PigSAFE) and weaned at 28 days post-farrowing (+/−2 days). Post-weaning piglets were maintained in litter groups in the PigSAFE pen. Analyses have been adjusted for sex both within and between litter as the only statistically significant covariate to play behaviour. Litter differences were observed in locomotor play in both the pre- and post-weaning stage (Pre: F_(6,76)_ = 5.51 P < 0.001; Post: F_(6,69)_ = 4.71, P < 0.001) and run (Pre: F_(6,76)_ = 4.96, P < 0.001; Post: F_(6,69)_ = 4.58, P < 0.001; the major element of locomotor play). Twenty eight% of the variance for a single observed animal in pre-weaning locomotor play and 26% of variance post-weaning could be attributed to the litter. There was no statistical evidence of differences in social play between litters at either stage with only 8% of pre-weaning variance, and 1% of post-weaning variance being attributable to the litter level. However non-harmful fighting (the major element of social play), showed strong evidence of litter differences in both periods (Pre: F_(6,76)_ = 2.38, P = 0.037; Post: F_(6,69)_ = 2.60, P = 0.025), and was the only aspect of the play behaviour to correlate between the pre- and post-weaning periods (r = 0.765, df = 5, P = 0.045). On average play increased post-weaning. Litters showed a ‘litter weaning effect’ by differing in their locomotor play behavioural response to weaning, measured as the change in locomotor play behaviour from pre- to post-weaning (F_(6,70)_ = 5.95, P < 0.001). These results generally confirm previous work showing litter differences in aspects of play behaviour in both the pre and post-weaning period. However, there was no consistency in litter differences between pre- and post-weaning periods in the categories of play behaviour with the exception of non-harmful fighting. We demonstrated a ‘litter weaning effect’ where litters respond as a ‘unit’ to weaning in terms of their locomotory play behaviour. In general these results add further support to the use of play as a sensitive welfare indicator in neonatal pigs.

## Highlights

• Litter differences in play behaviour occur pre- and post-weaning.
• More than 25% of the variance in locomotor play was attributable to the litter.
• 'Non harmful fighting’ was unique in showing consistency pre- to post-weaning.
• Litters differ in their locomotor play response to weaning (‘litter weaning effect’).
• Suggests a ‘common factor’ at the litter level creating variation in locomotor play.

## 1 Introduction

Play behaviour remains a topic of considerable interest in the behavioural sciences (see Graham and Burghardt, 2010 for a recent review). Play has also been proposed as an indicator of animal welfare (e.g. Held and Špinka, 2011), partly on the basis of play being adversely affected by fitness challenges such as loss of nutrition (Muller-Schwarze et al., 1981) and injury (Berger, 1979). Conversely play also responds positively to nutritional supplementation (e.g. Sharpe et al., 2002). The general sensitivity of play to environmental conditions suggests that play has the characteristics of a ‘luxury’ or ‘elastic’ behaviour, only being performed when environmental conditions are ‘good’ and ‘proximate needs’ have been met (Lawrence, 1987).

Pigs present an excellent model of play behaviour. Play in pigs has been described in wild and domesticated species (*Sus scrofa*) (e.g. Frädich, 1974, Dobao et al., 1985, Pellis and Pellis, 2016), and generally has similarities to play found in other species of young mammal (e.g. Newberry et al., 1988). As with other species, play behaviour in pigs can be categorised into locomotor, object-directed and social play (e.g. Blackshaw et al., 1997). The behaviours that are recognised as play in pigs have some resemblance to adult behaviours (e.g. running; play fighting) but at the same time are recognisably different being performed in an exaggerated, energetic and repetitive manner (Newberry et al., 1988). Social play in pigs demonstrates some of the difficulties involved in defining play behaviour as fighting in young pigs can be rough and closely resemble real fighting (e.g. Šilerová et al., 2010).

The study of individual differences in behaviour has become commonly used as an approach to understanding the causes and consequences of behaviour (e.g. Bell et al., 2009). Despite this, few studies have examined individual consistency in play behaviour over time. For polytocous species such as the pig, there is the added complexity that variation in play behaviour can come from the individual or the litter levels. There are reports of consistent litter differences in play in cats (Martin and Bateson, 1985) and dogs (Pal, 2010), and more recently in mink (Dallaire and Mason, 2016). In previous work we have reported on within and between litter differences in the play of pre-weaned domesticated pigs (Brown et al., 2015). Half of the variation in play in our study was attributable to consistent differences over time between litters (50%), with considerably less (11%) arising from consistent differences over time between individuals within litters. In our study (unlike Dallaire and Mason, 2016) there was no evidence that these litter differences were associated with differences in general activity. We also reported a strong positive association between litter differences in play and physical growth.

Weaning under natural conditions is a complex process involving phased reductions in the receipt of maternal investment (e.g. Martin, 1984, Borries et al., 2014). Under experimental and practical conditions (e.g. on farm) weaning is often abrupt, occurring at relatively early developmental periods (e.g. Jarvis et al., 2008). In rodents it is known that early abrupt weaning can have long-term, potentially detrimental effects on social behaviour and anxiety (Shimozuru et al., 2007). In pigs there is much evidence that this abrupt and early weaning poses challenges in terms of development of the piglets’ gut and adaptation to solid food (e.g. Wijtten et al., 2011) and also through the physiological and behavioural responses of piglets to the psychological components of weaning (e.g. Weary and Fraser, 1995). Mason et al. (2003) found that there were individual differences in vocalisation responses to weaning that correlated with piglet weight and teat choice; heavier piglets responded to weaning as a nutritional challenge (with ‘begging’ calls) with lighter piglets responding more as if they experienced maternal separation (with ‘separation calls’). Given the sensitivity of play to environmental challenges (see above) it seems reasonable to anticipate that play might be a good indicator of weaning stress.

This study extended our previous research (Brown et al., 2015) to investigate whether litter differences in play existed in both the pre- and post-weaning period and how these litter differences associated with physical development over the weaning event. We hypothesised (a) that there are litter differences in play behaviour in the pig prior to and following weaning imposed at 4 weeks post-partum; (b) that these litter differences in play will reflect the relative changes in developmental trajectory from pre- to post-weaning as measured by physical growth. Confirmation of these hypotheses would further indicate the usefulness of litter differences as an approach to the study of play and provide evidence of play behaviour as a potential indicator of development and welfare.

## 2 Material and methods

### 2.1 Ethical review

All work was carried out in accordance with the U.K. Animals (Scientific procedures) act 1986 under EU Directive 2010/63/EU following ethical approval by SRUC (Scotland’s Rural College) Animal Experiments committee under ED AE 05-2015. All routine animal management procedures were adhered to by trained staff and health issues treated as required. All piglets were returned to commercial stock at the end of the study.

### 2.2 Animals and housing

Pre- and post-weaning behavioural observations were carried out on litters from seven commercial cross-bred dams (Large White x Landrace); the boar-line was American Hampshire. Litters were born within a 72 h time window. Eighty three piglets were used in the study. Litter size was not standardised and was dependent on biological variation (11–13 piglets surviving until weaning per litter in this study). Sex ratios were not standardised with percentage of males range 15%–75% (mean = 48%). Cross fostering was kept to a minimum and only performed where piglet welfare was considered at risk, at which point piglets were fostered off the trial sow and on to the recipient sow within 24 h of farrowing. Pre-weaning mortality was 2.5%, with no piglet losses beyond 48 h after birth.

The experimental animals were housed in the *Pig and Sow Alternative Farrowing Environment* (PigSAFE) pens (Baxter et al., 2015) from birth through to 8 weeks of age (4 weeks post-weaning). PigSAFE pens allow species-specific behaviours in both the sow and the piglets to be expressed (Baxter et al., 2015) by providing more space and the provision of straw (1 kg per pen per day approximately). All pens have barred sections in the dividing walls allowing sows and piglets to see and touch those in neighbouring pens. Sows were of parity one or 2 with no prior experience of PigSAFE pens. Temperature within the unit was automatically controlled at 20 °C from birth until 1 week old, then reduced to 18 °C from 1 week to weaning, in accordance to the Defra Code of Recommendations for the Welfare of Livestock (Defra, 2003). Additional heat was provided in the creep area via under-floor heating at 30 °C. At weaning room temperature was increased to 22 °C with the creep temperature allowing additional heat source. Artificial lighting was maintained between the hours of 0800–1600 with low level night lighting ensuring Defra codes were adhered to. Piglet management included weighing at birth and a standard iron injection at day 3 post-partum. No teeth clipping, tail docking or castration was performed. Piglets were ear tagged for identification at both birth and at weaning. Sows were fed according to a standard feeding curve prior to farrowing (Baxter et al., 2015) and fed to appetite from approximately 2 days post-farrowing. Sows and piglets had ad libitum access to water. At weaning sows were removed from the pen and returned to the sow house while piglets were weighed and vaccinated against Porcine Circoviral Disease (PCVD). Litters remained intact in PigSAFE pens until the end of the study period (8 weeks of age) when they were moved to commercial farm stock. At approximately day 21 of age piglets were introduced to “creep feed” (Primary Diets DQ63P SL Silver pellets with no additional additives, AB Agri Ltd., Yorks, UK). Between 28 and 35 days of age piglets were gradually moved onto Primary Diets Prime Link Extra (pelleted, AB Agri Ltd., Yorks, UK). This was provided ad libitum post-weaning. Piglets were provided with additional drinkers post-weaning.

### 2.3 Piglet measures

Piglets were weighed within 24 h of birth. Piglets were subsequently weighed at days 5, 14 and 21 post-farrow, at weaning and when moved to farm stock at 8 weeks of age. For statistical purposes litter size pre-weaning was taken as the number of piglets that survived to weaning. No piglet losses occurred post-weaning. Piglet growth in the pre- and post-weaning periods are displayed as average daily gain (ADG). ADG was calculated as (end period weight-start period weight)/number of days and is presented in grams.

### 2.4 Recording of play behaviours

The animals were digitally recorded from birth in their home pen using Sony LL20 low light cameras with infra-red (RF Concepts Ltd, Belfast, Ireland) and a Geovision GV-DVR (Geovision GV-DVR, ezCCTV Ltd, Herts, UK). Two cameras were set up per pen, one at the rear and one at the front to provide maximal coverage. Piglets were not visible when in the far corner of the heated sleeping area, but could be seen at all other times. The observer was not present in the room during video recording. Pre-weaning observations occurred between the hours of 1030 and 1430 on days 5, 10, 14, 18, 21 and 24 post-farrowing with post-weaning observation days on days 4, 6, 8, 11 and 13 post -weaning. On observation days (between 0800 and 1000), piglets were numbered on the back with numbers corresponding to their randomly allocated post-farrowing ID’s using a black permanent marker. Cameras were set to record and video data analysed for the time period 1030–1430. The time period was chosen to commence after early morning husbandry and to extend for a period that would contain sufficient play bouts for analysis. The collected video material was continuously observed to identify play bouts, defined as episodes where at least one piglet was observed to engage in playful behaviour (see Table 1). Play behaviour for each individual piglet during these play bouts was then observed to identify specific behaviours using Noldus’ *The Observer XT 11* (Noldus Information Technology bv, Wageningen, The Netherlands) software package. Play behaviours were determined using an ethogram largely based on previous work in pigs (see Table 1); non-harmful fighting was included in the category of social play (Brown et al., 2015).

**Table 1:** Ethogram used for behavioural analysis with full descriptions and citations where categories are based on previous work. Behavioural categories are in bold and elements in regular font. Only those behaviours reported on have an expanded definition. Other behaviours that make up the play categories are mentioned within the category definition.

**Category**/Behavioural Element	Definition	References
**Locomotor Play**	Energetic movements with momentum including twirling of the body on a horizontal plane (pivot), jumping with two front feet or all four feet off the pen floor at one time (hop), dropping to the floor from a standing position (flop) and rapid forward movement (run).	Chaloupková et al. (2007), Newberry et al. (1988), Donaldson et al. (2002), and Bolhuis et al. (2005).
Run	Energetic running and hopping in forward motions within the pen environment. Often associated with excitability, using large areas of the pen, and occasionally coming into marginal/accidental contact with other piglets (e.g. nudge).	Chaloupková et al. (2007), Newberry et al. (1988), Donaldson et al. (2002), and Bolhuis et al. (2005).
**Social Play**	Energetic interaction between two or more piglets. Includes use of snout to gently touch another piglet’s body, not including naso-naso contact (nudge), using head, neck or shoulders with minimal or moderate force to drive into another piglet’s body (push), placing both front hoofs on the back of another piglet or sow (climb) and non-harmful fighting (as below).	Blackshaw et al. (1997), Bolhuis et al. (2005), Brown et al. (2015), Chaloupková et al. (2007), and Donaldson et al. (2002).
Non-harmful fighting	Two piglets mutually push and head-knock each other. A general mild intensity of the performed fighting behaviours and a lack of biting distinguish non-harmful fighting from potentially harmful fighting.	Brown et al. (2015)
**Object Play**	Animal manipulates an item or securely holds it in its mouth, energetically shaking it or carrying it around the pen.	Newberry et al. (1988)

Where more than one animal was observed starting a play bout simultaneously, the video was analysed for one animal and then rewound and analysed for the others. Play data were recorded as frequency counts. One observer completed all video analysis to remove any reliability issues relating to multiple observers.

### 2.5 Statistical analysis

Due to the high number of zeros the first observation day was dropped from the analysis. This led to five observation days in both the pre- and post-weaning periods. Frequency data was then totalled per piglet for each behaviour pre- and post-weaning across all five days. These count totals were square root transformed prior to statistical analysis in order to satisfy more closely the assumptions underlying the statistical methods applied. We analysed square root transformed frequency counts of the three play categories (locomotory, social, and object), and for running and play-fighting as the main behavioural elements comprising the locomotory and social play categories respectively (object play as a category had no constituent behavioural elements). As previously (Brown et al., 2015), we addressed the statistical analysis of within and between litter differences in play in two ways. Firstly, we fitted a mixed model comprising both fixed and random effects using the REML algorithm. This approach broadens the inference from the specific litters studied to the population of litters. The random effects part of the model comprised two terms: litter and piglets within litters, providing estimates of variance components for these two sources of variation. Thus, the variance component for litter is an estimate of the variance for the population of litters from which the seven observed in the study were a sample. The fixed effects part of the model included sex except for models for change between pre-and post-weaning where sex was dropped after testing for a possible effect. In addition, other potential covariates (see Table 2) were fitted individually with sex in order to assess whether there was statistical evidence of the need to adjust for these covariates when considering litter effects and litter differences in play behaviours. Sex was the only covariate where there was statistical evidence of an effect in the model (see Table 2). From the estimated variance components, it was possible to estimate the percentage of the variance for a single observed animal’s total attributable to the litter. Secondly, as in Brown et al. (2015; see also Martin and Bateson, 1985 for a similar approach) we used Analysis of Variance (with sex as a covariate) to compare litters in a fixed effects model with one value per individual (being the transformed value of the total over observation days within the pre- or post-weaning period). We tested for litter differences over the pre- and post-weaning periods separately. In addition, we tested the effect of weaning on play behaviour by calculating the change in behaviour as the post-weaning transformed frequency counts minus the pre-weaning transformed frequency counts per individual. We compared these estimates of the change in play behaviour between litters using both mixed models (REML) and ANOVA as with the other analyses. Pearson’s correlations of REML adjusted means (adjusted for sex in all comparisons excluding those regarding change from pre- to post-weaning, as there was no evidence of an effect of sex on these changes) were estimated in order to compare behaviours across the pre- and post-weaning periods and to assess potential associations with physical, measurable factors (e.g. ADG). Unless a significance level is stated, the term “significance” throughout the paper refers to statistical significance at the 5% level. Statistical analysis was carried out using Genstat (18th Edition).

**Table 2:** REML covariate analysis for the pre- and post-weaning periods. Covariates are listed across the top of the columns and behaviours analysed down the side. F and P values are given for each covariate for each behaviour. Due to its strong effect, sex was kept in the model for pre- and post-weaning but not for the change between pre- and post-weaning. Each other covariate was tested individually after adjusting for sex. Sex was observed to have a significant effect on social play and non-harmful fighting pre- and post-weaning, and on locomotor play and run post-weaning **(bold)**. There was evidence of an effect of sow parity on change in social play. No other covariates were found to affect behaviour in this model.

			Sex	Litter Size	Sow Parity	ADG pre-wean	ADG post-wean	Wean age
PRE	Locomotor	F	2.61	1.00	2.96	0.06	–	–
		P	0.110	0.364	0.146	0.802	–	–
	Social	F	20.22	0.33	3.09	2.03	–	–
		P	<0.001	0.590	0.139	0.161	–	–
	Object	F	0.15	0.94	1.78	0.34	–	–
		P	0.701	0.378	0.239	0.565	–	–
	Run	F	2.30	1.17	4.92	0.05	–	–
		P	0.133	0.329	0.077	0.832	–	–
	Non-harmful fighting	F	45.36	0.17	0.95	0.08	–	–
		P	**<0.001**	0.695	0.375	0.775	–	–

POST	Locomotor	F	4.47	0.23	0.23	–	1.10	1.32
		P	**0.038**	0.653	0.654	–	0.297	0.304
	Social	F	42.14	1.16	2.32	–	0.01	0.04
		P	**<0.001**	0.331	0.187	–	0.924	0.852
	Object	F	0.43	1.97	0.00	–	1.46	4.17
		P	0.513	0.221	0.967	–	0.232	0.103
	Run	F	4.32	0.015	0.23	–	1.39	1.59
		P	**0.041**	0.716	0.652	–	0.243	0.265
	Non-harmful fighting	F	40.57	1.27	2.04	–	0.02	2.30
		P	**<0.001**	0.311	0.212	–	0.896	0.193

CHANGE (pre- to post-wean)	Locomotor	F	0.64	1.05	2.77	0.75	0.46	0.21
		P	0.425	0.353	0.157	0.388	0.501	0.666
	Social	F	1.92	1.00	6.42	2.20	0.00	0.84
		P	0.170	0.364	0.054	0.149	0.992	0.402
	Object	F	0.90	5.98	1.27	0.47	0.02	0.70
		P	0.347	0.059	0.313	0.499	0.888	0.442
	Run	F	0.77	0.99	3.94	0.67	0.38	0.29
		P	0.384	0.365	0.104	0.417	0.540	0.611
	Non-harmful fighting	F	0.01	0.35	0.50	1.99	0.73	0.00
		P	0.910	0.581	0.513	0.183	0.399	0.967

## 3 Results

### 3.1 Litter differences in play counts pre- and post-weaning

From the mixed model analysis sex was the only covariate for which there was evidence of an association with any of the behaviours analysed (see Table 2). As such all results reported have been adjusted for sex only, with the exception of those regarding change pre- to post-weaning (what we have referred to as the ‘litter weaning effect’) as there was no evidence of an effect of sex on this variable. In both the pre-and post-weaning period males were observed to perform more social play behaviours (Pre- Male mean = 3.79, female mean = 2.53, SED = 0.281: Post- Male mean = 4.30, female mean = 2.39, SED = 0.295) including non-harmful fighting (Pre- Male mean = 2.41, female mean = 1.19, SED = 0.181: Post – Male mean = 2.90, female mean = 1.57, SED = 0.209). Post-weaning females were observed to perform more locomotor behaviour (Male mean = 3.39, female mean = 4.02, SED = 0.297) including running (Male mean = 3.27, female mean = 3.87, SED = 0.286), although this did not reach statistical significance in the pre-weaning period.

Litter differences were observed during the pre- and post-wean periods in the category locomotor play (Pre: F_(6,76)_ = 5.51 P < 0.001; Post: F_(6,69)_ = 4.71, P < 0.001) but not in categories of social or object play (see Table 3). In the category of locomotor play the largest proportion of behaviour (91.0%) was in the form of “run” while in the category social play the largest proportion (41.1%) was in the form of “non-harmful fighting”. The behaviour element run also differed between litters in both the pre- and post-wean periods (Pre: F_(6,76)_ = 4.96 P < 0.001; Post: F_(6,69)_ = 4.58, P < 0.001. Fig. 2). Contrary to the social play category result, there was statistical evidence that the social behaviour “non-harmful fighting” also differed between litters in both the pre- and post-wean periods (Pre: F_(6,76)_ = 2.38 P = 0.037; Post: F_(6,69)_ = 2.60, P = 0.025. Fig. 2). The variance component analysis for an individual animal (see Table 4) attributed 26% of the variance in pre-weaning running, and 11% of pre-weaning non-harmful fighting to the litter. Similarly, 25% of the variance in post-weaning run behaviour, and 13% of post-weaning non-harmful fighting behaviour was attributable to the litter of origin. These values are similar at the category level for locomotor play (% variance attributable to the litter: Pre:28%, Post:26%) but are lower for the social play category (Pre:8%, Post:1%).

**Table 3:** Fixed effects analysis of litter differences in the frequencies of behavioural categories (in bold) and elements (not bold) pre- and post-weaning. Variance ratios and probability values are adjusted for sex within litter as a covariate in the model.

		**Locomotor**	**Social**	**Object**	Run	Non-harmful fighting
Pre-weaning	Variance Ratio	5.51	1.99	2.16	4.96	2.38
	P	**<0.001**	0.077	0.056	**<0.001**	**0.037**

Post-weaning	Variance Ratio	4.71	1.05	2.12	4.58	2.60
	P	**<0.001**	0.400	0.061	**<0.001**	**0.025**

**Table 4:** Variance components analysis showing the estimated percentage contribution of litter (Litter%) to the variance of an individual observed animal in behavioural categories (in bold) and elements (not bold). Each cell in rows labelled ‘Litter’ and ‘Piglet in litter’ contains the variance component for that factor. Total variance in the model can be calculated as the sum of the variance components for litter and piglets within litter. Pre- and post-weaning variance estimates have been calculated after adjusting for sex. The Litter% value is calculated as the variance component for Litter/Total variance.

		**Locomotor**	**Social**	**Object**	Run	Non-harmful fighting
Pre-weaning	Litter	0.695	0.130	0.044	0.574	0.075
	Piglet in litter	1.771	1.547	0.44	1.664	0.636
	Litter%	28.2	7.8	9.0	25.6	10.6

Post-weaning	Litter	0.529	0.013	0.078	0.473	0.114
	Piglet in litter	1.528	1.634	0.75	1.419	0.769
	Litter%	25.7	0.8	9.4	25.0	12.9

CHANGE (pre- to post-)	Litter	1.250	0.1100	0.071	1.050	0.000
	Piglet in litter	2.755	3.220	1.060	2.584	1.518
	Litter%	31.2	3.3	6.3	28.9	0.0

**Fig. 1:**
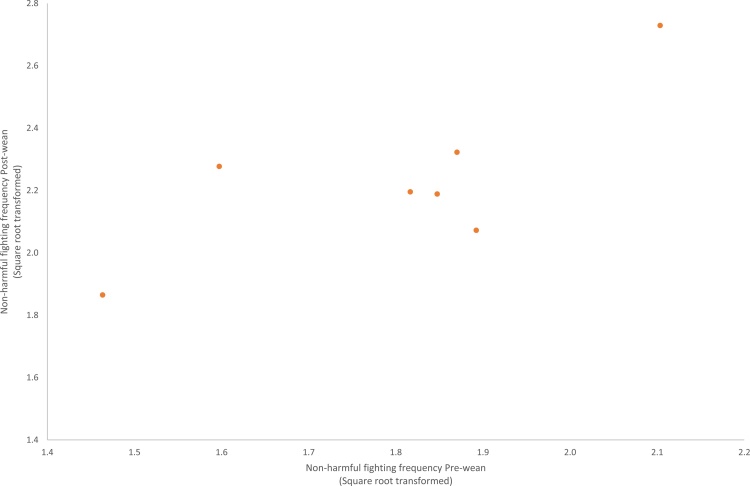
Litter means for the frequency per animal of non-harmful fighting events in the pre-weaning period against the post-weaning period. Litter means have been adjusted for sex (REML analysis). Frequency data has been square root transformed.

Analysis performed on litter means (transformed frequencies) from the REML analysis adjusted for sex found no statistical evidence of an association between pre- and post-weaning behaviours over the play categories or the behavioural elements. The exception was non-harmful fighting where there was a positive correlation between pre- and post-weaning stages at the litter level (r = 0.765, df = 5, P = 0.045; Fig. 1).

**Fig. 2:**
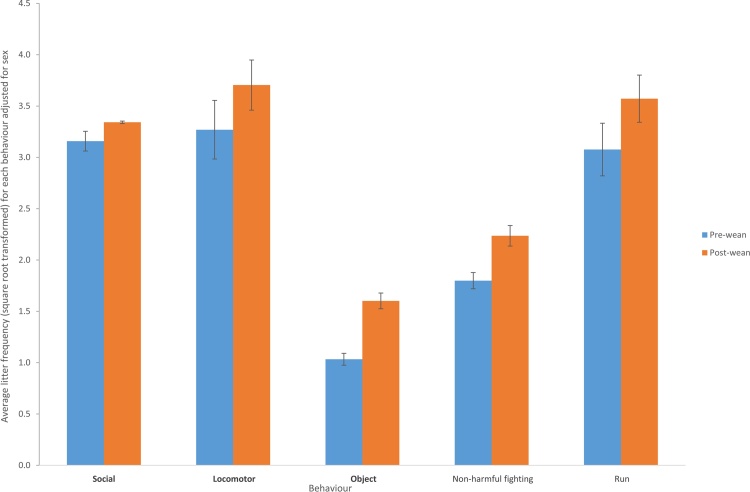
Mean transformed frequency values for the pre- (blue) and post-wean (orange) periods by behavioural category (in bold) and element (not bold). Frequency values shown are the means across all litters after adjusting for sex. Error bars show the standard errors of the litter means. Behaviours measured are observed to occur more frequently post weaning.

### 3.2 The effect of weaning

**Fig. 3:**
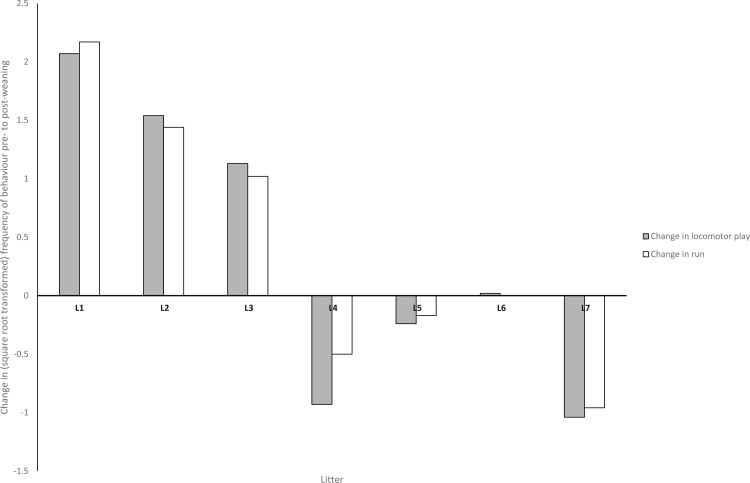
Change in play behaviour pre- to post-weaning for litters 1–7 (L1–L7). Values for each litter are extracted from the ANOVA table of means. Grey bars show the change in locomotor play pre- to post-wean by litter. White bars show the change in running behaviour pre- to post-wean by litter. Litter 6 shows no change in frequency of behaviour pre- to post-weaning.

Overall expression of play behaviour was greater in the post-weaning period compared to the pre-weaning period (Fig. 2). The effect of weaning on play behaviour was calculated as the difference in frequency between the pre- and post-weaning using the pre-weaning frequencies as the baseline.REML covariate analysis did not find any statistical evidence of an association between any of the covariates tested (sex, litter size, sow parity, average daily gain and weaning age) and the change in behaviour pre- to post-weaning (Table 2). Litters were observed to differ in their response to weaning in the change (pre- to post-weaning) in locomotor play (F_(6,70)_ = 5.95, P < 0.001; Fig. 3). Three litters displayed a reduction in locomotor play pre- to post-weaning, three litters displayed an increase in locomotor play pre- to post-weaning and one litter did not change its frequency of locomotor play between the two developmental stages. There was no statistical evidence that litters differed in their change in social or object play between pre- and post-weaning.

**Fig. 4:**
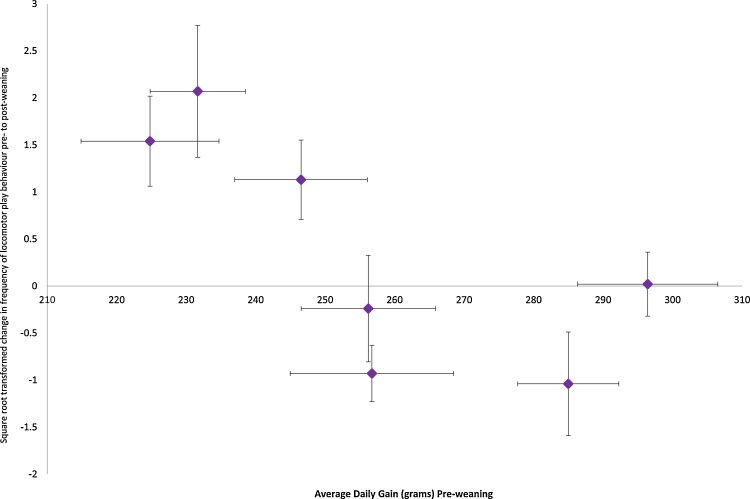
Change in locomotor play behaviour pre- to post-wean against average daily gain (ADG; grams) in the pre-weaning period. Data-points are the average per litter, square root transformed. Horizontal error bars give the standard error of the mean for ADG, vertical error bars give the standard error of the change in locomotor play.

There was no statistical evidence of an effect on growth during the post-weaning period as a result of the observed weaning effect, however growth during the pre-weaning period was found to show a trend towards a negative association with the change in locomotor play from pre- to post-weaning (r = −0.731, df = 5, P = 0.062) (Fig. 4).

## 4 Discussion

In a previous study (Brown et al., 2015) we observed litter differences in play behaviour in piglets during the pre-weaning period when raised in a free farrowing system. In this study, our aim was to confirm this finding and to determine if these litter differences persisted in the early post-weaning period. We also aimed to investigate how litter differences in play responded to changes in developmental trajectory across weaning as measured by physical growth.

The results generally confirm those of our previous work (Brown et al., 2015) showing litter differences in aspects of play behaviour in both the pre- and post-weaning period. We were able to corroborate our previous statistical evidence of litter differences in locomotor play, running (as the main component of locomotor play) and in non-harmful fighting (the major behavioural element of social play) in both the pre- and post-weaning periods. We did not find litter differences in object directed or social play categories. In this study litter differences appeared stronger post-weaning, which could be related to the increased levels of play post-weaning (see below).

Given that we had previously shown pre-weaning litter differences in play (Brown et al., 2015) and Rauw (2013) found that litter of origin affected play in a test of playfulness in post-weaned pigs, it was reasonable to expect a correlation between pre- and post-weaning litter differences. However, we found no evidence of consistency between pre- and post-wean periods in any of the categories of play behaviour and the behavioural element run, at the litter level. We did find non-harmful fighting (see Table 1 and Brown et al. (2015) for a definition) to positively correlate across the developmental stages. Pigs are relatively unique in that their non-harmful play fighting lacks the restraint that is observed in most species; that is, piglets appear to play to win and do not appear to self-handicap during play fighting (Pellis and Pellis, 2016). It has previously been suggested play fighting in pigs is therefore a practical opportunity to develop hostile manoeuvres with relatively reduced risk in a way that other species who show true restraint are not able to (Smith, 1982, Pellis and Pellis, 2016). As such, it could be that the performance of play fighting and specifically non-harmful fighting is under different motivational control than that of other play behaviours such as running or object manipulation. As a general point as far as we are aware this is the first study to investigate the consistency of litter differences in play before and after weaning, with the exception of non-harmful fighting (D’Eath and Lawrence, 2004), so we are limited in the comparisons we can make with the wider literature.

The observation that overall play increased post-weaning confirms the previous result of Donaldson et al. (2002) who observed higher levels of locomotor play in piglets at days 3 and 5 post-weaning relative to the pre-weaning period. They suggested that this could be related to space allowance as their piglets were moved to larger play pens, or an age effect as locomotor play has previously been shown to peak at around 4–5 weeks of age (Newberry et al., 1988). In this study we removed the sow rather than move the piglets from the farrowing environment, and the removal of the sow would in effect have given the piglets more space available for play (also observed by E Baxter when the sow uses the PigSAFE feeding stall pre-weaning, pers. comm.).

As with previous studies males expressed more social play behaviours (including non-harmful fighting) while females showed more locomotor play behaviours (Brown et al., 2015, D’Eath and Lawrence, 2004, Rauw, 2013). Locomotor play such as running and pivoting has previously been suggested as an indicator of positive emotion in pigs (Reimert et al., 2013) and calves (Krachun et al., 2010). In our previous study (Brown et al., 2015) we found that run appeared to be a good proxy for total play overall. It is interesting to note that the variance in locomotory play behaviours could be attributed to litter to a higher degree than those of the social play behaviours. This may suggest that whatever factor is responsible for driving play behaviour at the litter level (e.g. contagion, space allowance, nutrition and maternal care as discussed below) has a greater influence on the locomotor play behaviours than the social play behaviours, and that social play may be more dependent on the characteristics of the individual piglets. Work on individual differences in social interactions in piglets would be useful to develop this further.

Abrupt and early weaning is a stressful event (reviewed in Weary et al., 2008) that has behavioural, physiological and neuroendocrinological effects on young animals (reviewed in Campbell et al., 2013 and Enríquez et al., 2011). Here we report that variation between litters was greater than within litters in terms of the change in locomotory play over the pre- and post-weaning periods, in other words that litters responded as a unit to weaning in their locomotory play. This might suggest an effect of contagion where individuals within the litter affect the behaviour of others increasing the variability between litters. We cannot discount this but for it to be a complete explanation, it would also need to account for the reductions in play (pre- to post-weaning) seen in some litters and we know of no work suggesting such a negative contagion effect on play. Furthermore, in our previous work we did not find evidence that contagion was a strong influence on litter differences in play (Brown et al., 2015). Another explanation is of a litter level factor (or factors) which results in litters showing consistent gradation in terms of increasing or decreasing their locomotor play post-weaning relative to the pre-weaning period. This would suggest that changes in locomotor play pre to post-weaning are a sensitive indicator of the impact of weaning at the litter level.

In terms of factors contributing to the litter weaning effect we did find a trend for the change in locomotory play pre- to post-weaning to associate with a high growth rate (ADG) pre-weaning at the litter level. One interpretation of this would be that piglets, which experienced better nutritional support from the sow pre-weaning and hence grew faster, were more negatively affected by the weaning process, as reflected by their greater reduction in locomotory play pre- to post-weaning. While the number of litters in this study is small, this trend is somewhat supported by theories and observations on resource availability and play behaviour. The Surplus Resource Theory (Burghardt, 2005) predicts that greater resource availability will increase play levels and previous work in horses has shown that levels of play behaviour mirror maternal investment (Cameron et al., 2008) as measured by maternal change in body condition over the pre-wean period. Play has also been shown to be adversely affected by reduced nutrition (e.g. deer fawns; Muller-Schwarze et al., 1981: dairy calves; Krachun et al., 2010) while being positively affected by supplementation (e.g. Meerkats; Sharpe et al., 2002). Changes in locomotor play pre to post-weaning may therefore be a sensitive indicator of the relative loss of maternal nurturance at weaning at the litter level but further work, and a greater sample size, would be required to confirm this or to investigate other possible associations.

## 5 Conclusions and implications

These results generally confirm previous work showing litter differences in aspects of play behaviour in both the pre- and post-weaning period. We estimated that over 25% of variation in locomotor play pre- and post-weaning was attributable to the litter level, while less than 8% of the variation in social play pre- and post-weaning was attributable to the litter. We also found strong evidence that sex had an effect on the play behaviour observed with male rich litters showing more social play and female rich litters more locomotory play confirming previous work. Although we found no evidence of consistency in litter differences between pre- and post-weaning periods in the categories of play behaviour, we did observe litter differences in the locomotory play behaviour response to weaning which we have referred to as the ‘litter weaning effect’. We propose that this litter weaning effect suggests a common factor (or factors) operated at the level of the litter to create consistent variation in the response of locomotory play to the weaning challenge. As one potential explanation of the weaning effect we found a trend for a relationship between pre-weaning ADG and the locomotory play behaviour response to weaning. This could suggest that litters that were thriving pre-weaning experience a greater ‘check’ at weaning which was reflected in the change in locomotory play. However further work is required to confirm this. In general these results add further support to the use of locomotor play as a sensitive welfare indicator in neonatal pigs.

## Conflict of interest

None.
